# The Action of Nicotine on the Circulation

**Published:** 1891-09

**Authors:** 


### THE ACTION OF NICOTINE ON THE CIRCULATION.

Drs. Wertheimer and Colas have recently completed an elaborate experimental investigation as to the action of nicotine on the heart and circulation (Revue Internationale de Bibliographie, May 25, 1891.] They find—

First. As regards the heart, destruction of accelerator nerves, or of the extrinsic accelerator centres, does not prevent the production of increased frequency in the cardiac action, as a result of the two phases of poisoning with nicotine. As a consequence, the action of the poison is exercised exclusively on the intrinsic ganglia.

Second. The increased excitability of the cardiac muscle in poisoning by nicotine is, in the author's opinion, evidenced by the fact that excitation of the apex leads to the production of a series of pulsations and not a single beat only.

Third. As regards the action of nicotine on the vascular system, when a simultaneous registry is made of the general blood pressure and of the volume of one of the abdominal organs, the spleen, or the kidneys, it is found that the former diminishes at the moment where the latter increases. This state of affairs is followed by a second phase, during which the fall of pressure coincides with an increase in the volume of the organ. These two successive modifications of pressure are independent; the first being dependent on the constriction of the small splanchnic vessels, the second on their relaxation. While the blood pressure is increasing, the mucous membrane of the lips and of the tongue becomes the seat of an intense congestion from the excitation of the vaso-dilators of that region. After the complete destruction of the spinal cord, the injection of nicotine still determines an increase of blood pressure, which may amount to as much as twelve centimetres. Further, if the nerves of one side of the tongue or the lips are divided, the congestion of these parts produced by nicotine is quite as marked as in the normal side. The authors believe that this experiment tends to demonstrate the existence of purely peripheral vaso-dilator and vaso-constrictive ganglia.—*Therapeutic Gazette.*

